# Edible Insects: Consumption, Perceptions, Culture and Tradition Among Adult Citizens from 14 Countries

**DOI:** 10.3390/foods13213408

**Published:** 2024-10-25

**Authors:** Raquel P. F. Guiné, Sofia G. Florença, Cristina A. Costa, Paula M. R. Correia, Luísa Cruz-Lopes, Bruno Esteves, Manuela Ferreira, Anabela Fragata, Ana P. Cardoso, Sofia Campos, Ofélia Anjos, Nada M. Boustani, Elena Bartkiene, Cristina Chuck-Hernández, Ilija Djekic, Monica Tarcea, Marijana Matek Sarić, Zanda Kruma, Malgorzata Korzeniowska, Maria Papageorgiou, Leticia González Árias, Maša Černelič-Bizjak, Emel Damarli, Vanessa Ferreira, Emre Bayraktaroğlu, Fatmanur Ozyurek Arpa

**Affiliations:** 1 CERNAS Research Centre, Polytechnic University of Viseu, 3504-510 Viseu, Portugal; sofiaflorenca@outlook.com (S.G.F.); amarocosta@sc.ipv.pt (C.A.C.); paulacorreia@esav.ipv.pt (P.M.R.C.); lvalente@estgv.ipv.pt (L.C.-L.); bruno@estgv.ipv.pt (B.E.); 2 Health Sciences Research Unit: Nursing (UICISA: E), Polytechnic University of Viseu, 3504-510 Viseu, Portugal; mmcferreira@gmail.com (M.F.); sofiamargaridacampos@gmail.com (S.C.); 3 CIDEI-IPV Research Centre, Polytechnic University of Viseu, 3504-510 Viseu, Portugal; afragata@estgl.ipv.pt (A.F.); a.p.cardoso@esev.ipv.pt (A.P.C.); 4 CERNAS-IPCB, Research Centre for Natural Resources, Environment and Society, Polytechnic University of Castelo Branco, 6001-909 Castelo Branco, Portugal; ofelia@ipcb.pt; 5 Faculty of Business and Administration, Saint Joseph University, Beirut 1104 2020, Lebanon; nada.mallahboustany@usj.edu.lb; 6 Department of Food Safety and Quality, Lithuanian University of Health Sciences, 47181 Kaunas, Lithuania; elena.bartkiene@lsmuni.lt; 7 Tecnologico de Monterrey, The University for Obesity Research, Monterrey 64849, Mexico; cristina.chuck@tec.mx; 8 Department of Food Safety and Quality Management, Faculty of Agriculture, University of Belgrade, 11000 Belgrade, Serbia; idjekic@agrif.bg.ac.rs; 9 Department of Community Nutrition and Food Safety, GEP University of Medicine Pharmacy Science and Technology of Targu Mures, 540139 Targu Mures, Romania; monica.tarcea@umfst.ro; 10 Department of Health Studies, University of Zadar, 23000 Zadar, Croatia; marsaric@unizd.hr; 11 Faculty of Food Technology, Latvia University of Life Sciences and Technologies, LV 3001 Jelgava, Latvia; zanda.kruma@llu.lv; 12 Faculty of Food Science, Wroclaw University of Environmental and Life Sciences, 51-630 Wrocław, Poland; malgorzata.korzeniowska@upwr.edu.pl; 13 Department of Food Science and Technology, International Hellenic University, 57001 Thessaloniki, Greece; mariapapage@ihu.gr; 14 BALAT Research Group, Faculty of Veterinary Medicine, University of León, 24071 León, Spain; lgona@unileon.es; 15 Department of Nutritional Counseling–Dietetics, Faculty of Health Science, University of Primorska, 6320 Izola, Slovenia; masa.cernelic@fvz.upr.si; 16 Research and Development Center, Altıparmak Food, Çekmeköy, 34782 İstanbul, Turkey; emel.damarli@balparmak.com.tr; 17 Department of Nutrition, School of Nursing, UFMG—Federal University of Minas Gerais, Belo Horizonte 30130-100, Brazil; vanessa.nutr@gmail.com; 18 Department of Nutrition and Dietetics, Faculty of Health Sciences, Istanbul Medipol University, Beykoz, 34815 İstanbul, Turkey; ebayraktaroglu@medipol.edu.tr (E.B.); fozyurek@medipol.edu.tr (F.O.A.)

**Keywords:** insect consumption, consumer habits, COVID-19, gastronomy, exotic food, questionnaire survey

## Abstract

Although edible insects (EIs) are encouraged as a sustainable source of protein, their consumption is not as generalised as other types of food that are internationally accepted. While in some regions of the world, EIs are part of the gastronomic and cultural traditions, in other regions, people are not so receptive to this type of food, and some people even express some disgust towards it. Hence, this research focused on the habits of the participants regarding the consumption of insects as well as their perceptions about EIs being or not a part of the local culture or gastronomic patrimony. A questionnaire survey was implemented in fourteen countries (Brazil, Croatia, Greece, Latvia, Lebanon, Lithuania, Mexico, Poland, Portugal, Romania, Serbia, Slovenia, Spain, and Turkey), and globally, 7222 adult participants responded to the questionnaire. SPSS software (version 28) was used to process the data and carry out chi-square tests and Factor Analyses (FA). The obtained results showed significant differences between countries for all the questions included in the survey, either those regarding the habits of the participants or their opinions about the facts linked with EI tradition or cultural aspects. It was found that participants from Mexico consume EIs more than in all other countries and that strong motivations that would lead to consumption among those who do not consume include curiosity and food shortage. The solution obtained with FA considering the ten statements of the scale consisted of two factors: F1—Culture and Tradition of EIs (α = 0.675) and F2—Acceptance of EIs (α = 0.614). In conclusion, the consumption of EIs and the perceptions of people are highly variable according to geographic location and cultural environment.

## 1. Introduction

According to the United Nations’ 2024 report, the global population was 8,161,972,572 on the 1st of June 2024, and it is expected to surpass 8.5 billion by 2030 and 9.6 billion by 2050 [1]. This indicates that within 5 years, 400 million more people will require food resources, and within 25 years, this number will increase to 1.5 billion. Given that the food demand is projected to double by 2050, it is anticipated that agricultural lands may become insufficient, particularly leaving low-income countries vulnerable in terms of food security [2]. Despite the significant increase in population in recent years, there has been a notable decline in the number of deaths due to protein–energy malnutrition or famine. The substantial rise in the global population is being matched by an even greater increase in food supply [3].

The increase in food and beverage production brings concerns regarding greenhouse gas emissions, soil erosion of agricultural lands, pollution, and depletion of global water resources. Meat and dairy products, which are preferred due to their high protein content, are the most environmentally damaging food sources [4]. Humans need to consume proteins for survival; even if they do not use them as an energy source, proteins are essential for growth, development, adaptation, recovery, tissue formation, neuronal signalling, and the immune system [5]. Proteins obtained from alternative sources such as plants, microorganisms, and insects have garnered significant interest due to their lower environmental footprint and their potential to feed the growing global population [6].

In developed countries, approximately 10–15% of the energy in people’s diets is derived from proteins [5]. Edible Insects (EIs) are considered the best solution to meet the globally increasing protein demand [2,7,8] while being more environmentally friendly, emitting fewer greenhouse gases [9]. Additionally, insect farming requires significantly fewer resources, such as land, water, and feed, compared to traditional livestock farming [10]. Additionally, insects can be tended on organic waste, further lowering input costs and contributing to a circular economy [11]. This cost-effectiveness is particularly relevant in regions with limited resources or where food insecurity is a concern, making insect farming a viable and scalable solution for meeting the increasing global demand for protein.

While EIs are considered a source of protein, they are also rich in energy, vitamins, minerals, and fatty acids. The nutritional content of EIs varies depending on species, genus, gender, and maturity [12]. One hundred grams of caterpillars can meet the average daily vitamin requirements of humans [12]. Contrary to popular belief, EIs are considered to pose less risk of zoonotic diseases compared to mammals and birds, as they are taxonomically more distant from humans. Nevertheless, depending on the conditions of preparation, collection, and breeding, EIs are known to carry risks related to allergens, harmful microorganisms, mycotoxins, heavy metals, parasites, and pesticides [2]. In reality, even in societies where EIs are considered a discomfort food product, insect-derived food additives such as Carmine and Lac can be consumed [13].

EIs are consumed as a part of traditional cuisine in many parts of the world, particularly in southern or tropical regions. However, despite their nutritional content, entomophagy, meaning consumption of EIs, is often perceived as non-edible in Western societies due to associations with diseases [8,14]. Although there is growing interest due to environmental concerns, entomophagy remains at low levels in many countries. Cultural disgust towards EIs, the limited availability of insect-based foods, and the lack of regulation for their use as food and feed are the main reasons for this. Socio-cultural and psychological factors also influence people’s perceptions of EIs. Familiarity with foods, taste, flavour, and appearance is important when choosing new foods since these are expected to influence the acceptability of EIs in societies where they are not traditionally consumed [9].

The aim of this study was to investigate possible differences between countries in the habits of participants, especially regarding the consumption of EIs, and also assess how the participants feel about a number of issues related to EIs being or not part of the cultural environment or gastronomic patrimony in the cultures of the countries involved in the study. The research hypotheses were: (1) Consumption of EIs and the perception of their cultural acceptance significantly varies across different geographic regions and are influenced by local cultural traditions; (2) Regions with EIs as cultural heritage will report higher consumption, while those without will show lower acceptance and more negative perceptions; (3) Motivations for consuming EIs among non-consumers will likely centre on curiosity or sustainability. By pursuing the answers to these questions, it will be possible to gather information about consumption habits and perceptions of citizens of different geographies and sociocultural environments, and this might be very useful in the future to better address possible strategies to incentivise consumption of EIs where they are not yet much consumed.

## 2. Materials and Methods

### 2.1. Instrument

The collection of data was achieved through a questionnaire survey, developed and validated [7] in the ambit of the EiSuFood Project (for additional information about the project you can visit the website: https://raquelguine.wixsite.com/eisufood, accessed on 15 September 2024). The questionnaire was developed respecting all ethical principles, such as those of the Declaration of Helsinki, and submitted to the Ethics Committee of the Polytechnic University of Viseu, who gave positive approval with reference 45/SUB/2021. The questionnaire was translated into all the native languages of the participating countries, following a back-translation methodology, and it was accessible online (on the Google Docs platform) following invitations shared by email and social media. Only adult participants (aged 18 years or over) were allowed to answer the questionnaire because after giving informed consent, they accessed the questions to participate in the survey. Each participant was able to stop answering the questionnaire at any time and could choose not to submit their answers.

The questionnaire was structured in different parts, and those used for this article were Part I—Sociodemographic data (six questions), Part II—Characterisation of participants’ habits (nine questions), and Part III—Culture and Tradition (ten questions for which the participants had to express their agreement on a central five-point Likert scale: 1 = strongly disagree, 2 = disagree, 3 = no opinion, 4 = agree, and 5 = strongly agree) [15]. The questionnaire is given as Supplementary Material.

### 2.2. Data Collection

The sample was a convenience sample of type snowball. Nevertheless, despite it not being a probabilistic sample, a reference for the minimum number of participants was estimated as indicative. To calculate the sample size, some assumptions had to be previously established:

- Target half of the adult population;
- Consider a 90% confidence interval;
- Apply a z-score equal to 1.645;
- Consider the power of the test of 5%, which corresponds to a minimum acceptable probability of 5%, to prevent type II errors [16,17].

Considering the assumptions mentioned above and an infinite population, the recommended number of participants will stabilise at 271; therefore, this minimum number should be guaranteed in each participating country [18,19]. As such, a target was established at the beginning of data collection to obtain a minimum of 300 responses in each country.

The data were collected in 2021 in 14 countries: Brazil, Croatia, Greece, Latvia, Lebanon, Lithuania, Mexico, Poland, Portugal, Romania, Serbia, Slovenia, Spain, and Turkey. Globally, a total of 7222 adult participants were obtained.

We focused on selecting countries that could engage with entomophagy, ensuring respondents had relevant knowledge or experience. While the distinction between highly developed, developing, and low-income countries and the presence of traditional versus new entomophagy is relevant, our initial aim was to understand perceptions across countries to better design future research, being inclusive. The selection of which countries to participate in the project initially envisaged the inclusion of all types of countries, i.e., countries where entomophagy is culturally accepted and countries where it is not. The project EiSuFood initially included 18 countries, but it was verified that in some of them, the partners were not able to collect a minimum reasonable of answers to the questionnaire, and as such, because they failed to deliver the data for the project, they had to be excluded. Unfortunately, the excluded countries were Cape Verde, Morocco, Nigeria, and Colombia, which were precisely countries where eating insects is more part of the cultural tradition. As such, the final sample of countries included a great majority of Western or European countries and two countries where there is some entomophagy in some of their regions: Mexico and Brazil.

### 2.3. Data Analysis

To analyse the data software, IBM^®^ SPSS Statistics, Version 28, (Armonk, NY, USA) was used. A Factor Analysis (FA) was used to analyse the ten scale items, i.e., the ten statements used in the questionnaire. The FA was obtained using the Principal Components and Varimax rotation method. The number of components to extract was determined according to the criterion of Eigenvalues greater than one. Indicators of suitability, such as the Kaiser–Meyer–Olkin (KMO) measure of sample adequacy and Bartlett’s test, were used prior to the analysis [20]. The reference values considered for KMO indicate that values ≥ 0.5 are acceptable, but higher values are desirable until the maximum of 1, which would be optimal [21]. In the FA, the variables with loadings lower than 0.4 (absolute value) were excluded from the factors [22,23]. The measure of internal consistency was assessed using Cronbach’s alpha (α), which was calculated for each of the factors obtained with FA [20,24]. As a reference for internal consistency assessment, the following intervals were used: acceptable consistency α ∈ [0.5; 0.7], good consistency α ∈ [0.7; 0.8], and very good consistency α ∈ [0.8; 1] [25,26,27].

To analyse possible differences between countries, crosstabs with a chi-square test were used. The Cramer’s coefficient (V) was calculated to assess the strength of the associations between the variable country and the different variables tested. As a reference for the Cramer’s V coefficient, the following values were used: V ≈ 0.1—weak association, V ≈ 0.3—moderate association, and V ≈ 0.5 or more—strong association [28]. A level of significance of 5% was considered in all tests.

## 3. Results

### 3.1. Sociodemographic Characterisation of the Sample

**Figure 1:**
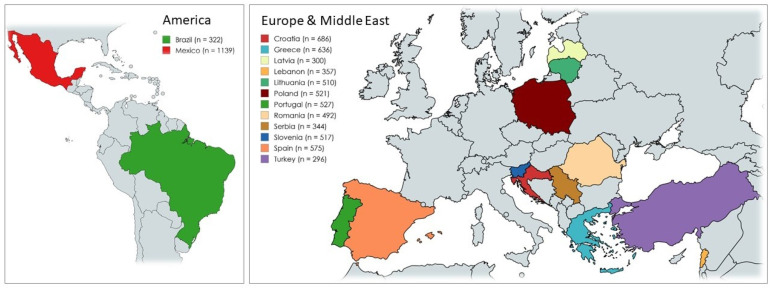
Participants in the survey according to country (*n* = 7222).

Figure 1 shows the geographical location of the countries, with 11 countries situated in Europe, one in the Middle East (Lebanon), and two on the American continent (Brazil in South America and Mexico in North America). Figure 1 also presents the number of participants in each of the 14 countries, summing to a total of 7222 participants. The highest number of participants was obtained in Mexico (*n* = 1139) and the lowest in Turkey (*n* = 296).

**Figure 2:**
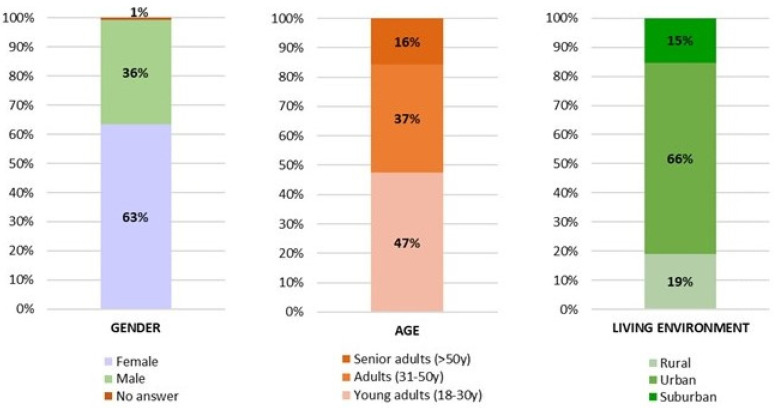
Distribution of the participants according to gender, age class, and living environment (classes of age: young adults, 18 to 30 years; adults, 31 to 50 years; senior adults, 50 years or over).

Figure 2 shows the distribution of the participants according to gender, age class, and living environment. More than half of the participants were female (63.5%), and concerning the age class, the most represented were young adults (47.4%) aged between 18 and 30 years old. Regarding the living environment, a majority lived in urban areas (65.6%), with lower percentages in suburban or rural areas (15.3% and 19.1%, respectively).

**Figure 3:**
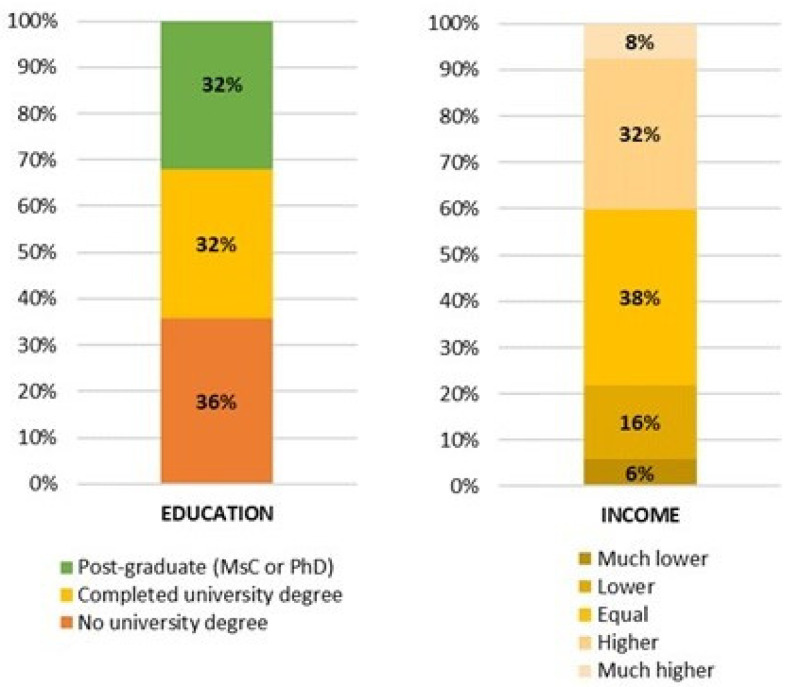
Distribution of the participants according to education and income.

Figure 3 presents the participants according to education level and household income. The distribution according to education level was relatively even, with 35.8% of the participants having an education level below university, 32.3% having completed a university degree, and 31.9% having completed post-graduate studies—master’s or doctorate degrees. With respect to income, most participants reported a household income similar to the average income in their own countries (38.1%).

**Figure 4:**
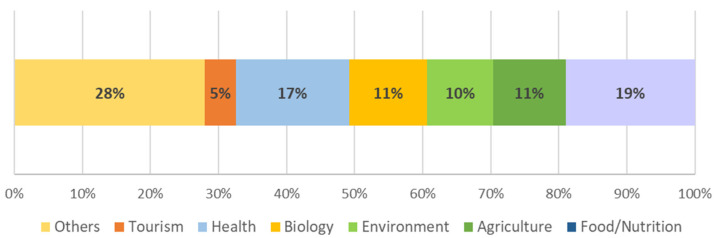
Professional activities of the participants.

The professional activities of the participants are presented in Figure 4. Considering the subject of the research, some categories were preselected and presented to the participants to choose from. The participants could identify their professional activities linked with more than one option, for example, agriculture plus environment. From the participants who answered this question with one of the given options, a higher number responded Food/Nutrition (*n* = 2269), followed by Health professionals (*n* = 1991), and with lower representativeness appear professionals in the area of Tourism (*n* = 544).

### 3.2. Characterisation of the Participants’ Habits

Table 1 presents results for four of the questions about the habits of the participants related to eating in restaurants, travelling abroad, and the type of food consumed in those circumstances. Since the data collection for this survey was undertaken in 2021, still under the influence of COVID-19, the participants were asked to consider their pre-Covid habits in their responses. For question QH-1, about the frequency of eating in restaurants, the most frequent answer considering the whole sample was ‘sporadically’ (between once per week and once per month), chosen by 2435 participants, followed by ‘seldom’ (less than once per month) with *n* = 2133 participants. Significant differences (*p* < 0.001) were found between countries for this question, but the association was weak (V = 0.165).

For the group of questions QH-2 (Table 1) about the type of food preferred when eating out in restaurants, the participants across the global sample indicated firstly ‘traditional’ (traditional food from my country, *n* = 3650), then ‘fast-food’ (*n* = 2538), followed by ‘ethnic food’ (typical from foreign countries, *n* = 2029). The option ‘fast-food’ was the first choice only in Turkey, but received a considerable number of votes from all other countries as well. In Croatia and Lithuania, the first option chosen by participants was ’grilled food/barbecue’ (*n* = 425 and *n* = 228, respectively), and in Brazil, the first choice was ‘health’ (*n* = 128). Significant differences (*p* < 0.001) were found between countries for all these questions, with strong associations between the variables (values of Cramer’s coefficient varying from V = 0.435 for option d to V = 0.620 for option h).

With respect to question QH-3 (Table 1) about the frequency of travelling abroad, the most frequent response was ‘rarely’ (about once per year) (*n* = 3200 participants of the global sample). Countries where the participants travel less, with the highest number of responses for option ‘never’ included Brazil (*n* = 193), Mexico (*n* = 670), and Turkey (*n* = 177). On the other hand, countries where people travel more include Slovenia, Greece and Poland (*n* = 103, *n* = 72, and *n* = 66 participants, respectively, chose option ‘often´). Significant differences (*p* < 0.001) were found between countries with a moderate association (V = 0.292).

The last question in Table 1, QH-4, about the type of food consumed abroad, clearly indicates that the participants want to consume food typical from the country they are visiting (*n* = 3265 participants, overall), with this being the first choice, regardless of the country of origin of the participants. Nevertheless, significant differences were also observed with a weak association (*p* < 0.001, V = 0.234)

Table 2 shows the results obtained for the questions aimed at investigating to what extent the participants considered that the COVID-19 pandemic changed their habits. For question QH-5.a (Change in the frequency of eating out), although most participants (*n* = 2326) considered that COVID-19 resulted in a high change, a high number of participants continued with the same frequency of eating out (*n* = 2046). The differences between countries were significant, with a moderate association (*p* < 0.001, V = 0.300).

Regarding the frequency of travelling abroad (question QH-5.b in Table 2), similar results were obtained as for the previous question, i.e., most participants reported a high change (*n* = 3254), but many did not register any change (*n* = 2521), which may have included those who did not use to travel before COVID-19. The differences between countries were significant, with a moderate association (*p* < 0.001, V = 0.257).

Considering the frequency of ordering prepared food to consume at home (question QH-5.c in Table 2), most of the participants declared they did not notice any change (*n* = 2695) or just a slight change (*n* = 2627), but significant differences (*p* < 0.001) were observed between countries with a weak association (V = 0.202), and for example, in Mexico, most of the participants declared they had noticed a big change in their frequency of ordering food (*n* = 482).

For questions about changes observed in the type of food consumed, food shopping practices, and food safety concerns (Questions QH-5bd, e, and f in Table 2), in all cases, most of the participants declared that they did not notice changes due to COVID-19 (number of participants who reported no changes were *n* = 3905, *n* = 3142 and *n* = 3237, respectively, for the three questions). However, significant differences were observed between countries in all three questions (*p* < 0.001), with moderate associations (V varying from 0.244 to 0.306).

Table 3 presents the results for questions that related specifically to the consumption of EIs. Regarding question QH-6 (Have you ever eaten insects as culinary preparations, as snacks or other derived products?), a great majority of the participants replied negatively (*n* = 5283 participants, considering the global sample), but the number of participants who had already consumed EIs was highest in Mexico (*n* = 755), which is a large country where consuming insects is traditional in some regions. The differences between countries were significant, with a moderate association (*p* < 0.001, V = 0.380).

Regarding question QH-7 (If you have never eaten insects, would you consider eating them?), a high number of respondents admitted they would not eat EIs (*n* = 2875), but also a considerable number admitted they might eat them (*n* = 2145). Again, in Mexico, the highest number of participants (*n* = 340) replied they would eat EIs, regardless of the form (whole insects and derived foods).

With respect to the motivations that could lead people to consume EIs (question QH-8, Under which motivations would you consume EIs?), the most frequent options were for curiosity (*n* = 3684) and in case of food scarcity (*n* = 2894). Options linked to culinary applications of EIs or their nutritive richness were factors much valued by Mexicans (*n* = 441 and *n* = 600, respectively). Significant differences were found between countries (*p* < 0.001) for all motivations, and the associations were strong (values of V varying from 0.528 for motivation a., curiosity, to 0.682 for d., culinary).

The last question in Table 3 relates to the frequency of consumption of EIs (QH-9, If you consume EIs, how often do you eat them as culinary preparations, snacks or other derived products?). The results indicate that the great majority consume EIs very rarely (once per year) (*n* = 1749), even in Mexico (*n* = 407). The differences between countries were significant, with a weak association (*p* < 0.001, V = 0.203).

### 3.3. Perceptions About Cultural and Traditional Aspects Related to Edible Insects

Ten statements were used to investigate the perception of the participants about cultural and/or traditional aspects related to EIs in the different countries. Table 4 presents the results for the global sample, i.e., considering all countries together. Considering that the middle point of the scale (score 3) is a neutral opinion, a high percentage of the participants chose score 3 for most of the statements except for two, with percentages of score 3 varying from a minimum of 27.4% to a maximum of 46.9. This indicates that the participants found it difficult to express an opinion of disagreement or agreement with the statements presented to them.

For S1, ‘Entomophagy is a dietary practice that consists in the consumption of insects by humans’ (not considering those who chose score 3), a high percentage of responses were in agreement, i.e., scores 4 (22.9%) and 5 (25.3%), indicating that the participants know what entomophagy is. Statement S3, ‘There are thousands of species of insects that are consumed by humans in the world’, obtained a high percentage of positive responses (38.5% for score 4 and 21.3% for score 5), revealing that even though the participants do not consider EIs a food traditionally from their country, they know about the variability of EIs that can be consumed by humans. For S8, ‘Insect consumption is seasonal, so it varies according to the time of the year’, the percentage of participants on the agreement side was a little higher (21.2% for score 4 and 7.1% for score 5) than for disagreement (8.8% and 16.0% for scores 1 and 2, respectively); these results reveal that a high number of people consider that insects are seasonal, which is particularly true for those collected in the wild, but not necessarily for those that are reared in farms.

For S2, ‘Insects are considered a traditional food in my country’, a high percentage of participants chose scores of disagreement (64.5% for score 1 and 15.2% for score 2), showing that EIs are not a traditional food globally, in the set of 14 countries included in the research. The results for S6, ‘Insects are part of the gastronomic culture of most countries in the world’, show a slightly higher percentage of participants on the disagreement side (12.5% for score 1 and 25.8% for score 2) than on the agreement side (22.9% for score 4 and 9.5% for score 5). This indicates that a high number of participants recognise that EIs are consumed in many countries, even if they are not consumed in their own country.

Regarding S4, ‘Consuming insects is characteristic of developing countries’, there was a trend to disagree (15.7% score 1 and 25.0% score 2), revealing that the participants did not consider EIs as food consumed only in developing countries or characteristic of more poor communities. With respect to S7, ‘In some countries the tradition of eating insects is decreasing because of the “Westernization” of diets’, a high percentage of participants agreed that although being traditional in some areas of the globe, EIs are being somehow neglected due to other foods imported from Western societies (30.4% for score 4 and 12.1% for score 5). For S9, ‘There are obstacles to consumers’ acceptance of edible insects in Western countries’, the results indicate a clear trend to agree (32.1% and 25.4% for scores 4 and 5, respectively), indicating that factors such as lack of familiarly, neophobia, or disgust may lead to rejection of EIs in Western countries.

For S5, ‘Insects are present in events related with religious rituals’, although the percentages of responses on the disagreement side were a little higher (14.5% for score 1 and 19.0% for score 2), there were also some participants who agreed that EIs could be a part of food in some religious festivities (16.6% for score 4 and 4.9% for score 5). For the related item S10, ‘Insects can be associated with traditional festivities and celebrations’, the participants showed a higher level of agreement (24.2% and 9.3% for scores 4 and 5, respectively), considering EIs part of the festivities and celebrations, but not necessarily those of religious nature.

Although the previous results in Table 4 are indicative of a trend in the global set of countries, it is important to see possible differences between the countries. Therefore, the results of the percentages of the scale scores obtained for all ten statements, separately by country, are shown in Table 5. The results indicate significant differences between countries for all ten statements (*p* < 0.001 in all cases), with associations varying from low (V = 0.154 for S3) to strong (V = 0.414 for S2).

According to the results in Table 5, the highest stronger agreement (score 1) with Statement S1 (Entomophagy is a dietary practice that consists of the consumption of insects by humans) was observed for Spain (44.9%) and Mexico (38.4%). For S2 (Insects are considered a traditional food in my country), most participants disagreed, and the strongest disagreement (score 1) was observed for more participants from Slovenia (90.3%), Croatia (87.2%), Spain (87.1%), and Poland (84.0%). For S3 (There are thousands of species of insects that are consumed by humans in the world), the participants were mostly on the agreement side, and maximum agreement (score 5) was highest in Mexico (41.7%), followed by Brazil (28.0%). For S4 (Consuming insects is characteristic of developing countries), the trend was to have a higher percentage of participants disagreeing, and score 1 was highest for Turkey (29.7%), Lithuania (23.9%), Spain (21.7%), and Serbia (20.9%). Regarding statement S5 (Insects are present in events related to religious rituals), also with a trend to disagree for most participants, score 1 was prevailing for Croatia (28.9%) and Spain (28.2%). For item S6 (Insects are part of the gastronomic culture of most countries in the world), more participants disagreed than agreed, with percentages of score 1 highest in Portugal (23.9%) and Spain (23.8%). For S7 (In some countries, the tradition of eating insects is decreasing because of the “Westernization” of diets), the participants tended to agree, with percentages of score 5 highest in Mexico (29.4%) and Lebanon (26.3%). For S8 (Insect consumption is seasonal, so it varies according to the time of the year), the participants were more on the agreement side, with percentages of score 5 highest in Mexico (24.3%) and Lithuania (9.4%). For statement S9 (There are obstacles to consumers’ acceptance of edible insects in Western countries), a high level of agreement was observed, particularly for Mexico, Spain, and Brazil, with percentages of score 5 of 40.6%, 39.3%, and 38.5%, respectively. Finally, for S10 (Insects can be associated with traditional festivities and celebrations), the highest percentages of score 5 were obtained for Mexico (25.5%) and Lebanon (15.7%).

### 3.4. Factor Analysis

The ten statements were submitted to FA to identify a possible grouping structure and to evaluate internal consistency. The correlation matrix between the ten variables, corresponding to the ten items of the scale, showed some associations between the variables, and this was confirmed by Bartlett’s test, which was significant (*p* < 0.001), thus implying the rejection of hypothesis H0 “The correlation matrix is equal to the identity matrix”. The value of KMO obtained was 0.844, which can be considered good [21]. According to the anti-image matrix, there are no values of the Measure of Sampling Adequacy (MSA) under 0.5, which is indicative that none of the variables should be excluded from the analysis at this stage. The values of the MSA varied from a minimum of 0.805 for statement S9 to a maximum of 0.869 for statement S8.

Using FA with PC extraction and Varimax rotation and considering the eigenvalues higher than 1, a solution with two factors that explained 43.0% of the variance was obtained (Table 6). Some communalities were higher than 0.5 (S1 = 0.505, S6 = 0.519, and S9 = 0.572). The first factor (F1) explained 23.9% of the variance and gathered six variables more linked with cultural and traditional aspects associated with EIs, and was named CTEI (Culture and Tradition of EIs). The second factor explained 19.1% of the variance and included four variables more related to the acceptance of EIs as food, so it was named AEI (Acceptance of EIs). According to the values of THE loading coefficients, item S6 (about EIs being part of gastronomic culture) was the one contributing more strongly to the factor F1 (loading = 0.718), while item S9 (about obstacles to consumption) was the one contributing more to the factor F2 (loading = 0.755). Both factors had an acceptable internal consistency [20] based on the values of Cronbach’s alpha (α = 0.675 for F1 and α = 0.614 for F2).

**Figure 5:**
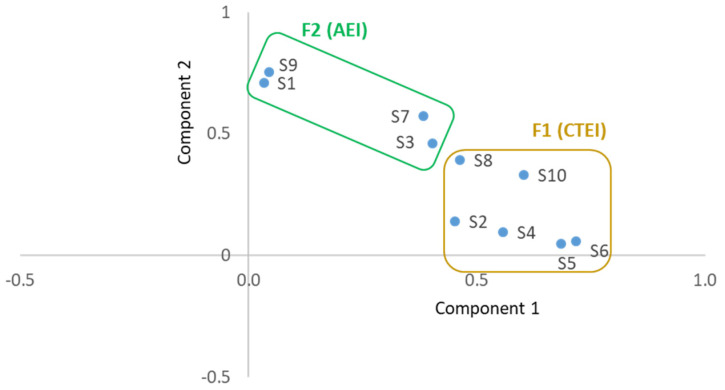
Factor Analysis component plot for solution obtained with Varimax rotation (AEI—Acceptance of EIs, CTEI—Culture and Tradition of EIs).

Figure 5 is a graphical representation of both factors considering the rotated space in the Cartesian coordinates system defined by both components of the FA. The graph shows the grouping structure that resulted from the FA, separating the two groups of variables, Factor F1 (CTEI) and Factor F2 (AEI).

## 4. Discussion

With the global population increasing, the demand for foods that are both nutritious and less harmful to the environment has become more pressing [29]. In this context, the consumption of EIs, an alternative source known to cause less damage to the ecosystem, stands out as a food source [30]. Many countries have already integrated EI consumption as part of their cultural life [31]. However, in some countries, the search for sustainable food sources is evolving and spreading towards an increase in EI consumption and production [32]. This research explores potential differences between countries in terms of participants’ habits related to EI consumption. It also examines whether EIs are part of the cultural or gastronomic landscape in various regions.

### 4.1. Characteristics of the Sample

This work investigated consumption habits, attitudes (acceptance, rejection motivations), and perceptions of EIs. Related to these topics, multiple studies have been conducted to assess people’s attitudes and consumption habits towards EIs across countries [33,34]. For instance, Asian countries are regions that host a wide variety of ethnicities and different culinary cultures, and a study was conducted to explore the demand trend for alternative protein sources. Adult Singaporeans (*n* = 1224) (75% Chinese, 15% Indian, 10% Malay) participated in the study. As a result, it was determined that welfare insect-based products, food neophobia, and animal welfare are the two main determinants of avoidance [34]. Our research was conducted in 14 countries from 3 continents, summing to a total of 7222 participants (Figure 1). Most of the participants were young adults (47.4%) aged between 18 and 30 years old. The great majority lived in urban areas (65.6%) (Figure 2). A notable strength of the study is that most of the study consisted of young adults. In one of the qualitative studies conducted in this field, a previous qualitative study has shown that younger generations tend to be more open to alternative food sources. This openness is often attributed to the pervasive influence of media [35]. Similarly, in the present study, young adults were mostly reached through media use and access originating from social networks. The fact that the distribution of individuals according to their level of education is similar is an indication that the distribution of demographic characteristics of the study is well-balanced. As noted in the literature, formal education may also be an important factor in EI consumption and perspectives [36]. In the current study, the distribution according to education level is quite even. However, another study found no statistically significant relationship between education level and EI consumption tendency [37]. Individuals working in the field of food and nutrition are likely to have a high level of perception about sustainability issues [38]. Given that the majority of the participants in this study have ‘Food/Nutrition’ and ‘Health’ professional activities, it is expected that their literacy in ecology and sustainability was relatively high.

### 4.2. Characteristics of the Participants’ Habits

According to the results of our study, the frequency of eating out and the food preferred by people in different countries differ. Most of the participants prefer traditional foods when they eat out, except in Turkey. In Turkey, fast food ranks first. In a study conducted in the USA, similar to this study, it was found that people eat out 1–3 times a month [39]. In 2021, there were 20,782 restaurants in Brazil, 640,000 in Mexico, and 70,000 in Romania [40,41,42]. A cross-sectional study showed that people living in areas with a higher density of restaurants ate at restaurants more frequently [43]. A study conducted in Italy showed that the local population preferred more traditional foods when eating out, which is consistent with our study [44]. The fact that the food-service market in Turkey is dominated by global and local fast food systems with a rapidly rising trend and the working population’s desire to consume their lunches quickly [45] may have affected our results in this direction. The distribution of the number of restaurants according to the population and surface area of the countries may have caused the difference in eating-out habits. It is also thought that the difference in economic distribution between countries is also effective in this regard. The frequency with which the participants eat in restaurants can help explain some degree of familiarity and cultural background that could be related to the attitudes and acceptance of new foods, particularly EIs. If consuming EIs is a usual or traditional practice in a participant’s region, they are likely to view them more positively and consume them more frequently. On the other hand, participants from regions where EIs are not part of their habits may perceive them negatively and be less willing to try or accept them. The frequency of restaurants can be a motivator to try new foods, such as an incentive by chefs, even in regions where they are not traditionally consumed.

People may travel to try or discover different and new local foods. In addition to this phenomenon, which is called gastronomy, cuisine, or food tourism, it has been reported that people change their level of satisfaction with their travels according to the foods they try in the places they travel [46]. According to a study conducted in Poland, it was observed that 12% of people who purchased a holiday abroad made a reservation only with the breakfast option, despite the all-inclusive option, with the desire to try local dishes in regional restaurants, to get to know different restaurants and to eat meals made entirely from ecological ingredients [47]. However, it has also been reported that this effect may vary according to the duration of stay in the foreign country [48]. It was expected that consistent with the literature, the people participating in this study would mostly prefer local foods when travelling abroad.

Participants in the study stated that there was no change in their habits of travelling abroad and eating out as a result of COVID-19. Perhaps these included those who also ate out and travelled abroad rarely before COVID-19, and for that reason, no change was observed.

While participants in Mexico reported a higher change, most of the participants stated that their habit of ordering prepared food did not change during COVID-19. Sociodemographic differences affect the habit and amount of ordering prepared food [49]. With the increase in the time spent at home during COVID-19, the rate of food preparation and eating at home has also increased. However, restaurants where people can go have been subject to a number of restrictions and measures [50]. In this direction, contrary to our study, it is expected that the frequency of ordering food from outside will decrease. This may have been balanced by the fact that people seek diversity instead of constantly preparing and eating food at home. The COVID-19 pandemic has affected each country to a different degree and caused different measures to be taken among countries according to their management styles, cultures, technologies and the level of development of their healthcare systems [50]. This difference between the countries in our results may also be due to the variability of the measures taken.

The majority of the participants in the study had never eaten EIs before. When the countries in the study were compared, it was seen that participants in Mexico experienced EIs more than other countries. In a study conducted in China and Germany, it was found that 67.7% of the Chinese and 10.3% of the Germans had eaten insects before, although no difference was observed in the distribution of neophobia [51]. A study conducted with Polish adults also showed that 67.6% of them had never eaten insects before [52]. Our findings are compatible with the fact that Mexico, which has regions where insects are traditionally consumed, similar to China, differs from other countries.

Most of our respondents who had never eaten EIs before said they would not want to try it. When asked what might motivate people to eat EIs, the majority responded with curiosity and scarcity. Among those who consume EIs, most reported consuming it once a year, even in Mexico. In a study conducted in Europe, only a small proportion of those who had never eaten EIs before stated that they would try EIs [53]. The Chinese participants in the study by Hartman et al. (2015) were similar to the Mexican participants in our study in that they said they would consume EIs regardless of the form, whereas the majority of German participants preferred it to be processed if they had to eat it [51]. Curiosity was found to be the strongest factor in the motivation to try EIs, which is in line with a study conducted in the UK [54]. In another study conducted with Swedes, environmental sustainability and health were determined as the two most important factors [55]. Although 37.8% of respondents in a survey conducted in the Czech Republic had eaten EIs before, only 11.8% reported eating them regularly [53]. Although people may have tried EIs out of curiosity, environmental awareness, and other factors, eating habits are not easily changed. For this reason, people may not have regularly substituted the foods to which they were accustomed.

### 4.3. Participant’s Perceptions About Cultural and Traditional Aspects Related to Edible Insects

Considering that score 3 is a neutral opinion on the scale, a significant percentage of the participants chose this option. The percentages of score 3 varied from a minimum of 27.4% to a maximum of 46.9. This suggests that participants found it challenging to express an opinion of disagreement or agreement with the statements presented to them.

Entomophagy refers to the eating of insects for food. Although this statement is a definition of entomophagy, its inclusion in the study is significant because it helps assess participants’ understanding and knowledge of the concept, which could be unfamiliar or misunderstood by many. With this response, it is possible to have knowledge levels about how familiarity with the term might influence attitudes, openness, or disposition to accept insects as food. In the current study, a high percentage of responses were towards agreement, indicating that the participants knew what entomophagy is. Familiarity with this term may be due to many of the participants having a professional background in food or nutrition/health. Another multi-country study found that Norwegian consumers were more likely to accept insects as food than Portuguese consumers. For example, in a study conducted in Germany, 93% of 718 people who participated said that their familiarity with insects was eaten instead of meat when it was specific [56]. The highest strong agreement with the statement was observed for Spain (44.9%) and Mexico (38.4%). Entomophagy is traditionally common in certain regions of some of the countries where the study was conducted, so high levels of familiarity are to be expected. A high percentage of positive responses revealed that even though the participants did not consider EIs a food tradition in their country, they knew about the variety of EIs that can be consumed by humans. The issue of alternative food sources is frequently mentioned in the media due to issues such as its spread in social media and the academic community, as well as the increase in environmental problems on the agenda [57]. In addition, since there are many individuals in our sample who are involved in professions in the field of nutrition and health, it is expected that they will know this subject. As in the previous statement, the countries with the most knowledge on this question are on the agreement side, and the maximum agreement score was highest in Mexico (41.7%) followed by Brazil (28.0%). Since EIs are consumed in some parts of these countries, individuals are likely to be aware of the prevalence of this consumption even if they do not consume it themselves. One of the study’s results reveals that many people consider insects to be seasonal, which is particularly true for those collected in the wild but not necessarily for those reared on farms [58]. For S8 (Insect consumption is seasonal, so it varies according to the time of the year), the participants were more on the agreement side, with percentages of score 5 highest in Mexico (24.3%) and Lithuania (9.4%).

In general, EI consumption is a part of gastronomic culture in some countries [59]. In this study, participants agree that EIs are consumed in many countries, even if they are not consumed in their own country. When asked whether insects are considered a traditional food in their country, the strongest opposition was observed by more participants from Slovenia (90.3%), Croatia (87.2%), Spain (87.1%), and Poland (84.0%). The statement that insects are part of the gastronomic culture of most countries in the world was opposed by more participants, especially from Portugal (23.9%) and Spain (23.8%). Regarding the habits and perceptions of the participants, the results of this study might be influenced by a certain asymmetry between the Western countries where entomophagy is not traditional, which constituted the majority of the countries included in the study, and some countries where eating insects is part of the traditional gastronomic culture, which were included in the survey in minor number. This limitation occurred due to the difficulty in obtaining responses from most of the countries initially planned as having some entomophagic culture. The reasons why these countries were not able to deliver data might be related to lower literacy, lower economic status, and lower available resources for conducting the study.

While the ecological benefits of consuming EIs are well known, some consumers (especially those in Western countries) are opposed to adopting insects as a food source [60]. In some Latin American countries, EIs consumption has traditionally existed but has been on the decline as the countries have adopted a more Western-style diet. A high percentage of participants agreed that even though it is traditional in some areas, EI consumption is being neglected due to other foods imported from Western-style diets [54]. The results show that participants have an opinion about factors such as lack of familiarity, neophobia, or disgust that may lead to rejection of EIs in Western countries, especially participants from Mexico, Spain, and Brazil, with percentages higher than 35% in each of them. In a study conducted in a part of Brazil with a Western culture that is not familiar with insect consumption, 780 people participated in the study. The primary association with edible insects was disgust [61].

Also, the participants in this study do not consider EIs as food consumed only in developing countries or as characteristic of poorer communities. For the idea of the statement, a higher percentage of disagreed participants were from Turkey (29.7%), Lithuania (23.9%), Spain (21.7%), and Serbia (20.9%). Eating insects may contribute to many SDGs concerning the development status of countries, but it cannot be an indicator of a single level of development [62]. Multiple factors affect this situation. In particular, the consumption of crickets as food is influenced by various cultural and religious belief systems that affect people’s desire to include crickets in their diets [63]. In the current study, although some agreed with the statement that insects were involved in events related to religious rituals, the rate of those who disagreed was slightly higher. Also, with a trend to disagree, most participants were from Croatia (28.9%) and Spain (28.2%). For the related item ‘Insects can be associated with traditional festivities and celebrations’, the participants showed a higher level of agreement, considering EIs part of the festivities and celebrations, but not necessarily those of a religious nature, with the highest percentages obtained from Mexico (25.5%) and Lebanon (15.7%). For example, the South African tradition of insect-eating was heavily dependent on caterpillars, which were abundantly available in the wild and preferred as food over beef, severely affecting the market sale of beef [64]. People’s ideas and attitudes about EIs may vary depending on the dynamics of their socio-cultural environment.

Some of the strengths of this study are that it has a large sample size, it was conducted in 14 different countries, and its demographic distribution is somewhat balanced. This situation provided a comprehensive evaluation opportunity and created strong data. However, it also has some limitations. One of the limitations is that the data collected in the study were collected retrospectively from individuals. This method is based on the memory of the participants. Therefore, it may create a bias. One other limitation is related to the way the sample was selected, by convenience following a snowball approach by sending out invitations to fill in the questionnaire. In this way, the different sociodemographic variables considered were not evenly distributed across the sample, namely in terms of gender, with a much higher participation of women than men and more participants from Mexico than from other countries. Even though we are conscious of these possible limitations of the study, the fact that the data were obtained from many different countries and from a very high number of individuals allows us to have an interesting overview of the perceptions of the participants about EIs and their integration with different cultures.

## 5. Conclusions

The results obtained showed that among the countries considered in the study, there are significant differences in the habits of the adult participants engaged in the study in what concerns eating in restaurants and travelling abroad, particularly the frequency of eating in restaurants, the type of food preferred, how often the participants travel abroad, or what kind of food they prefer when eating in a different country than their own.

Concerning the consumption of EIs, again significant differences were observed between countries, with a prevalence of Mexican participants that consume insects compared to the other countries. For those who had not yet consumed insects, the disposition to try was high in Portugal, Poland, Slovenia, and Spain. The strongest motivations to consume insects were found to be curiosity or lack of food.

Regarding the perceptions of the participants about cultural and traditional aspects of EIs, significant differences were found between countries for all the ten statements included in the questionnaire. The ten items were submitted to a factor analysis that extracted two factors, the first with six statements, F1—Culture and Tradition of EIs, and the second with four statements, F2—Acceptance of EIs. Both factors had acceptable internal consistency based on the values of Cronbach’s alpha (α = 0.675 for F1 and α = 0.614 for F2).

In this work, a preliminary diagnosis of consumption and perceptions and a comparison of realities between the 14 countries included in this study was carried out, which was something that had not yet been done to this extent. This diagnosis might be useful in the future for policymakers to eventually design campaigns aimed at incentivising EIs and promoting their use as sustainable alternatives to other meat sources.

The future of research on edible insects holds great potential in areas such as innovative product development, culinary exploration, food nutrition, sustainability, and economic development. Studies should investigate their nutritional value and environmental advantages over traditional livestock. Developing new processing methods to make insects more appealing to consumers is also a promising line of research. Acceptance can be promoted through educational campaigns and culinary innovation by collaborating with known chefs. If successfully integrated into the food system, edible insects could become a key component of sustainable and nutritious diets for future generations.

## Figures and Tables

**Table 1:** Eating in restaurants and travelling abroad, considering the global sample and by country.

		Countries ^(1)^													
	Total	BR	HR	GR	LV	LB	LT	MX	PL	PT	RO	RS	SI	ES	TR
QH-1. How often do you eat in restaurants (please answer considering the pre-Covid situation)? ^(2)^															
(Number of participants who replied yes)															
Never	243	14	81	10	12	6	41	20	5	16	13	14	8	1	2
Seldom	2133	74	322	179	133	74	147	268	134	157	157	130	207	100	51
Sporadically	2435	74	180	236	106	136	148	386	196	172	155	119	204	244	79
Occasionally	1611	60	86	181	35	80	111	305	124	124	119	58	54	187	87
Frequently	623	63	12	27	12	50	57	139	48	42	40	16	31	29	57
Very often	175	37	4	3	2	11	6	21	13	16	8	7	13	14	20
Chi-square ^(3)^	*p* < 0.001; V = 0.165														
QH-2. When you go to restaurants, what type of food you prefer (pre-Covid)? (possibility to answer up to 3 options) ^(4)^															
(Number of participants who replied yes)															
	Total	BR	HR	GR	LV	LB	LT	MX	PL	PT	RO	RS	SI	ES	TR
a. Traditional	3650	48	423	375	143	137	216	842	220	287	171	176	116	318	178
b. Ethnic	2029	89	111	166	105	76	56	293	264	125	156	72	204	247	65
c. Regional	1977	87	160	193	66	86	73	335	183	161	156	70	149	184	74
d. Gourmet	1356	36	160	96	88	61	88	151	78	43	95	104	155	134	67
e. Fast-Food	2538	74	249	244	75	123	126	629	175	176	111	102	124	139	191
f. Day-Dish	1497	70	103	106	90	63	116	207	70	176	82	34	108	231	41
g. Healthy	1690	128	114	108	81	85	38	240	110	135	167	53	185	162	84
h. Vegan/Veg	603	39	11	29	24	40	35	32	116	51	51	17	97	48	13
i. Grill/Barb	2846	66	452	347	49	110	228	607	56	250	134	151	166	84	146
Chi-square ^(3)^	*p* < 0.001 for all nine options (a to i); V(a) = 0.501, V(b) = 0.564, V(c) = 0.547, V(d) = 0.435, V(e) = 0.493, V(f) = 0.556, V(g) = 0.558, V(h) = 0.620, V(i) = 0.548														
QH-3. How often do you travel abroad (please answer considering the pre-Covid situation)? ^(5)^															
(Number of participants who replied yes)															
	Total	BR	HR	GR	LV	LB	LT	MX	PL	PT	RO	RS	SI	ES	TR
Never	1926	193	237	141	21	54	52	670	58	156	50	27	10	80	177
Rarely	3200	112	291	297	139	189	268	355	273	273	210	163	209	338	83
Sometimes	1443	10	108	126	106	78	150	77	124	77	145	108	195	121	18
Often	650	7	47	72	34	36	40	37	66	21	87	46	103	36	18
Chi-square ^(3)^	*p* < 0.001; V = 0.292														
QH-4. When traveling abroad do you have a preference for the type of food you consume? ^(6)^															
(Number of participants who replied yes)															
	Total	BR	HR	GR	LV	LB	LT	MX	PL	PT	RO	RS	SI	ES	TR
Typical local	3265	136	234	359	177	65	133	397	284	273	280	147	269	409	102
My country	1107	39	253	83	33	88	136	58	33	72	68	67	79	40	58
International	1	0	1	0	0	0	0	0	0	0	0	0	0	0	0
No preference	883	13	66	84	47	79	159	92	44	35	85	53	65	42	19
Chi-square ^(3)^	*p* < 0.001; V = 0.234														

^(1)^ BR = Brazil, HR = Croatia, GR = Greece, LV = Latvia, LB = Lebanon, LT = Lithuania, MX = Mexico, PL = Poland, PT = Portugal, RO = Romania, RS = Serbia, SI = Slovenia, ES = Spain, and TR = Turkey. ^(2)^ Never = zero times per month, Seldom = less than once per month, Sporadically = between once per week and once per month, Occasionally = about once per week, Frequently = 2 to 3 times per week, Very often = 4 or more times per week. ^(3)^ Chi-square test: *p* = significance (level of significance of 5%), V = Cramer’s coefficient. ^(4)^ a. Traditional food from my country, b. Ethnic food (typical from foreign countries), c. Regional specialities, d. Gourmet food, e. Fast-food, f. Suggestion of the day/Readily prepared dish, g. Healthy food, h. Vegan/Vegetarian, i. Grilled food/Barbecue. ^(5)^ Never = zero times per year, Rarely = about once per year, Sometimes = about 2 times per year, Often = 3 or more times per year. ^(6)^ Typical local = Typical food of the country I am visiting, My country = Food as similar as possible to my own country, International = International food (types of food commonly spread around the world).

**Table 2:** Influence of COVID-19 on food habits, considering the global sample and by country.

		Countries ^(1)^													
	Total	BR	HR	GR	LV	LB	LT	MX	PL	PT	RO	RS	SI	ES	TR
QH-5.a). Change in the frequency of eating out?															
(Number of participants who replied yes)															
Not changed	2046	62	413	129	162	88	219	151	154	120	114	153	226	0	55
Little changed	2273	101	214	215	119	142	174	318	239	203	98	128	179	0	143
Much changed	2326	159	57	292	19	127	117	670	128	204	280	63	112	0	98
Chi-square ^(2)^	*p* < 0.001; V = 0.300														
QH-5.b). Change in the frequency of travelling abroad?															
(Number of participants who replied yes)															
	Total	BR	HR	GR	LV	LB	LT	MX	PL	PT	RO	RS	SI	ES	TR
Not changed	2521	148	382	160	46	91	131	632	114	184	65	93	122	179	174
Little changed	1438	45	138	84	60	127	121	87	185	99	124	80	159	104	25
Much changed	3254	129	164	392	194	139	258	420	222	244	303	171	236	285	97
Chi-square ^(2)^	*p* < 0.001; V = 0.257														
QH-5.c). Change in the frequency of ordering prepared food in the catering sector?															
(Number of participants who replied yes)															
	Total	BR	HR	GR	LV	LB	LT	MX	PL	PT	RO	RS	SI	ES	TR
Not changed	2695	84	406	229	133	75	150	223	209	204	198	144	272	264	104
Little changed	2627	131	212	260	86	201	203	434	180	188	148	109	157	210	108
Much changed	1893	107	66	147	81	81	157	482	132	135	146	91	88	96	84
Chi-square ^(2)^	*p* < 0.001; V = 0.202														
QH-5.d). Change in the type of food consumed?															
(Number of participants who replied yes)															
	Total	BR	HR	GR	LV	LB	LT	MX	PL	PT	RO	RS	SI	ES	TR
Not changed	3905	112	493	402	150	106	277	322	333	274	335	215	335	410	141
Little changed	2510	150	165	183	128	177	144	548	147	214	131	107	156	137	123
Much changed	800	60	26	51	22	74	89	269	41	39	26	22	26	23	32
Chi-square ^(2)^	*p* < 0.001; V = 0.244														
QH-5.e). Change in the food shopping practices?															
(Number of participants who replied yes)															
	Total	BR	HR	GR	LV	LB	LT	MX	PL	PT	RO	RS	SI	ES	TR
Not changed	3142	102	417	318	95	56	168	250	295	3142	267	186	261	384	79
Little changed	2779	151	219	240	135	209	210	477	182	2779	163	124	189	152	114
Much changed	1295	69	47	78	70	92	132	412	44	1295	62	34	67	36	103
Chi-square ^(2)^	*p* < 0.001; V = 0.260														
QH-5.f). Change in the food safety concerns?															
(Number of participants who replied yes)															
	Total	BR	HR	GR	LV	LB	LT	MX	PL	PT	RO	RS	SI	ES	TR
Not changed	3237	89	387	335	233	22	246	209	362	240	251	191	311	294	67
Little changed	2315	123	194	202	54	141	165	399	124	214	134	105	167	178	115
Much changed	1663	110	102	99	13	194	99	531	35	73	107	48	39	99	114
Chi-square ^(2)^	*p* < 0.001; V = 0.306														

^(1)^ BR = Brazil, HR = Croatia, GR = Greece, LV = Latvia, LB = Lebanon, LT = Lithuania, MX = Mexico, PL = Poland, PT = Portugal, RO = Romania, RS = Serbia, SI = Slovenia, ES = Spain, and TR = Turkey. Note: Inadvertently, no data were collected in Spain for question QH-5.a. ^(2)^ Chi-square test: *p* = significance (level of significance of 5%), V = Cramer’s coefficient.

**Table 3:** Consumption of edible insects, considering the global sample and by country.

		Countries ^(1,2)^													
	Global N	BR %	HR %	GR %	LV %	LB %	LT %	MX %	PL %	PT %	RO %	RS %	SI %	ES %	TR %
QH-6. Have you ever eaten insects as culinary preparations, as snacks or other derived products? ^(3)^															
Yes	1507	2.0	1.7	3.3	4.4	1.8	9.1	50.1	8.5	3.0	2.1	1.3	3.6	7.8	1.4
No	5283	5.0	11.6	10.6	4.0	4.8	6.8	6.4	6.9	8.5	8.3	5.8	8.4	8.1	4.9
Other	432	6.7	11.1	6.7	5.3	17.6	3.0	10.4	6.7	7.6	5.8	4.2	4.6	6.5	3.7
Sum	7222	Chi-square ^(4)^: *p* < 0.001; V = 0.380													
QH-7. If you have never eaten insects, would you consider eating them? ^(5)^															
No	2875	4.9	15.2	11.9	2.7	5.4	5.4	9.5	7.9	6.9	6.3	6.1	6.5	4.9	6.3
Maybe	2145	6.2	8.0	9.0	6.2	6.2	8.1	0.0	9.7	10.7	8.9	5.4	9.1	8.9	3.6
Yes to some	738	2.7	4.2	5.6	7.5	8.1	16.8	19.1	6.4	4.3	5.8	2.3	8.0	7.6	1.6
Yes to all	835	3.2	3.7	7.3	3.8	0.8	6.6	40.7	4.7	4.1	2.5	3.2	4.8	11.5	3.0
Sum	6593	Chi-square ^(4)^: *p* < 0.001; V = 0.280													
QH-8. Under which motivations would you consume EIs? (you may choose more than one option) ^(6)^															
a. Curiosity	3684	4.5	7.1	6.2	4.7	4.1	8.8	17.9	6.8	7.7	6.8	4.8	7.4	10.4	2.9
b. No food	2894	4.9	15.1	4.9	1.6	5.5	5.4	13.1	6.5	9.0	4.9	3.7	9.9	8.5	7.0
c. Sustainable	1062	6.0	4.0	8.1	3.4	7.9	7.8	1.2	9.5	11.5	7.1	4.7	11.7	13.9	3.2
d. Culinary	1227	3.7	3.9	4.1	3.3	7.3	8.4	35.9	4.8	3.3	1.9	2.9	6.5	9.7	4.3
e. Nutrition	1826	4.7	3.9	5.5	3.0	5.3	6.1	32.9	5.9	5.2	4.8	2.7	7.9	9.8	2.5
Sum	10,693	Chi-square ^(4)^: *p* < 0.001 for all five options (a to e); V(a) = 0.528, V(b) = 0.559, V(c) = 0.620, V(d) = 0.682, V(e) = 0.696													
QH-9. If you consume EIs, how often do you eat them as culinary preparations, snacks or other derived products? ^(7)^															
1 x/year	1749	0.9	5.9	8.1	2.7	18.0	2.1	23.3	7.2	1.7	8.9	2.5	2.9	3.0	12.9
2–3 x/year	448	0.9	0.4	4.2	0.4	5.8	0.0	65.0	4.9	2.7	4.9	0.9	0.2	2.0	7.6
1 x/month	163	0.0	0.6	5.5	1.2	8.0	0.0	47.2	2.5	2.5	16.6	1.2	1.2	1.8	11.7
1 x/week	49	0.0	0.0	16.3	0.0	8.2	0.0	30.6	2.0	0.0	16.3	4.1	0.0	2.0	20.4
2+ x/week	33	0.0	0.0	9.1	3.0	0.0	0.0	27.3	0.0	6.1	18.2	6.1	3.0	6.1	21.2
Sum	2442	Chi-square ^(4)^: *p* < 0.001; V = 0.203													

^(1)^ BR = Brazil, HR = Croatia, GR = Greece, LV = Latvia, LB = Lebanon, LT = Lithuania, MX = Mexico, PL = Poland, PT = Portugal, RO = Romania, RS = Serbia, SI = Slovenia, ES = Spain, and TR = Turkey. ^(2)^ Results expressed as percentages calculated in relation to the total number of responses for each option in each question. Note that the sum of answers in question QH-8 is more than the number of participants because the participants could choose more than one option. ^(3)^ Other = Don´t know/Don´t remember. ^(4)^ Chi-square test: *p* = significance (level of significance of 5%), V = Cramer’s coefficient. ^(5)^ No = Definitely not, Maybe = Possibly, Yes to some = Yes, but only derived foods that include insects (for example, hamburgers or biscuits), Yes to all = Yes, whole insects and derived foods. ^(6)^ a. Curiosity = Out of curiosity, b. No food = If there is a scarcity of food, c. Sustainable = To help preserve the planet, d. Culinary = Because of the gastronomic characteristics, e. Nutrition = Because of the nutritional properties. ^(7)^ 1 x/year = About one time per year, 2–3 x/year = About 2 to 3 times per year, 1 x/month = About one time per month, 1 x/week = About one time per week, 2+ x/week = Two or more time per week.

**Table 4:** Scores attributed for each of the ten statements, considering the global sample.

Code	Statements	% of Responses				
		Level of Agreement ^(1)^				
		1	2	3	4	5
S1	Entomophagy is a dietary practice that consists in the consumption of insects by humans	5.5	6.2	40.1	22.9	25.3
S2	Insects are considered a traditional food in my country	64.5	15.2	7.2	7.3	5.8
S3	There are thousands of species of insects that are consumed by humans in the world	4.4	8.3	27.4	38.5	21.3
S4	Consuming insects is characteristic of developing countries	15.7	25.0	34.6	19.1	5.7
S5	Insects are present in events related to religious rituals	14.5	19.0	45.0	16.6	4.9
S6	Insects are part of the gastronomic culture of most countries in the world	12.5	25.8	29.3	22.9	9.5
S7	In some countries the tradition of eating insects is decreasing because of the “Westernization” of diets	6.0	12.4	39.1	30.4	12.1
S8	Insect consumption is seasonal, so it varies according to the time of the year	8.8	16.0	46.9	21.2	7.1
S9	There are obstacles to consumers’ acceptance of edible insects in Western countries	5.9	9.3	27.4	32.1	25.4
S10	Insects can be associated with traditional festivities and celebrations	11.4	15.7	39.5	24.2	9.3

^(1)^ Values on a 5-poin Likert scale: 1 = strongly disagree, 2 = disagree, 3 = no opinion, 4 = agree, and 5 = strongly agree.

**Table 5:** Percentage of participants’ responses, according to country, for the ten statements.

Code ^(1)^ Stats ^(2)^	Score ^(3)^	Percentage of Responses Within Country													
		Country ^(4)^													
		BR	HR	GR	LV	LB	LT	MX	PL	PT	RO	RS	SI	ES	TR
S1	1	5.0%	8.9%	5.5%	2.3%	3.1%	5.5%	4.3%	5.0%	5.0%	7.3%	9.6%	3.7%	3.5%	9.5%
*p* < 0.001	2	3.4%	5.0%	7.5%	3.0%	2.5%	14.9%	4.8%	5.4%	5.3%	8.3%	11.3%	4.8%	4.3%	6.8%
V = 0.207	3	38.2%	46.6%	21.1%	71.7%	19.0%	22.2%	30.9%	49.0%	45.8%	37.2%	61.3%	60.3%	31.1%	64.2%
	4	19.9%	19.3%	47.5%	15.7%	37.5%	31.6%	21.6%	23.5%	19.1%	24.8%	10.2%	11.6%	16.2%	12.2%
	5	33.5%	20.2%	18.4%	7.3%	37.8%	25.9%	38.4%	17.1%	24.8%	22.4%	7.6%	19.5%	44.9%	7.4%
S2	1	53.1%	87.2%	79.6%	75.3%	57.7%	47.1%	6.0%	84.0%	79.3%	68.7%	79.4%	90.3%	87.1%	70.9%
*p* < 0.001	2	22.0%	8.2%	17.0%	23.0%	33.1%	32.5%	8.6%	13.1%	12.5%	19.1%	14.5%	6.0%	8.0%	19.9%
V = 0.414	3	14.9%	3.2%	3.1%	1.0%	6.2%	18.0%	15.0%	0.6%	5.7%	8.7%	4.7%	2.7%	2.3%	6.8%
	4	6.8%	1.0%	0.3%	0.0%	2.0%	2.4%	38.1%	0.8%	1.9%	2.6%	0.9%	0.6%	1.2%	1.4%
	5	3.1%	0.4%	0.0%	0.7%	1.1%	0.0%	32.3%	1.5%	0.6%	0.8%	0.6%	0.4%	1.4%	1.0%
S3	1	3.1%	7.4%	3.5%	2.7%	2.0%	5.9%	3.6%	3.8%	4.7%	5.5%	4.7%	2.1%	5.7%	5.1%
*p* < 0.001	2	5.3%	10.9%	8.8%	8.3%	2.8%	13.5%	5.3%	7.7%	9.7%	7.7%	9.6%	7.0%	13.9%	4.1%
V = 0.154	3	25.8%	30.3%	34.7%	33.7%	18.8%	27.6%	14.9%	23.8%	31.5%	29.1%	36.3%	40.4%	30.1%	17.2%
	4	37.9%	32.8%	41.2%	49.0%	57.4%	35.9%	34.5%	45.0%	37.2%	42.1%	36.9%	31.1%	30.4%	49.3%
	5	28.0%	18.5%	11.8%	6.3%	19.0%	17.1%	41.7%	19.6%	16.9%	15.7%	12.5%	19.3%	19.8%	24.3%
S4	1	19.3%	16.9%	14.5%	5.0%	6.2%	23.9%	11.9%	8.7%	17.5%	11.8%	20.9%	17.2%	21.7%	29.7%
*p* < 0.001	2	26.1%	21.6%	28.0%	34.7%	6.7%	34.7%	17.7%	23.7%	23.9%	31.3%	26.7%	23.6%	29.4%	33.8%
V = 0.167	3	35.7%	36.9%	38.1%	38.3%	26.9%	23.7%	38.4%	35.8%	37.2%	31.9%	40.7%	37.1%	29.0%	27.0%
	4	13.4%	19.2%	17.5%	20.0%	35.0%	12.4%	22.1%	28.3%	18.4%	20.1%	11.3%	18.0%	16.2%	7.4%
	5	5.6%	5.4%	2.0%	2.0%	25.2%	5.3%	9.9%	3.7%	3.0%	4.9%	0.3%	4.1%	3.7%	2.0%
S5	1	15.5%	28.9%	6.1%	6.0%	6.7%	15.9%	13.4%	6.2%	17.5%	11.6%	10.2%	14.3%	28.2%	11.8%
*p* < 0.001	2	21.4%	19.4%	12.7%	25.3%	5.9%	25.9%	19.2%	13.8%	22.0%	20.9%	17.2%	19.9%	24.2%	15.5%
V = 0.179	3	45.3%	39.7%	57.1%	54.7%	27.5%	38.2%	40.2%	48.5%	46.9%	44.3%	52.9%	51.8%	38.4%	54.7%
	4	12.4%	9.0%	20.8%	13.3%	40.9%	15.1%	18.2%	26.7%	11.2%	19.3%	17.2%	10.8%	8.0%	14.5%
	5	5.3%	3.1%	3.3%	0.7%	19.0%	4.9%	9.0%	4.8%	2.5%	3.9%	2.6%	3.1%	1.2%	3.4%
S6	1	16.5%	17.8%	13.7%	5.3%	6.7%	9.8%	6.0%	9.6%	23.9%	5.5%	7.0%	18.8%	23.8%	6.8%
*p* < 0.001	2	31.1%	34.0%	36.9%	33.7%	8.4%	16.3%	17.9%	33.1%	33.2%	4.9%	12.2%	34.3%	40.2%	19.3%
V = 0.236	3	32.9%	31.9%	33.8%	28.7%	22.1%	26.7%	29.1%	32.7%	29.2%	18.1%	34.3%	33.7%	25.4%	31.1%
	4	14.6%	11.5%	13.4%	29.0%	31.1%	30.0%	31.2%	22.1%	11.0%	49.2%	34.0%	10.9%	8.5%	33.1%
	5	5.0%	4.8%	2.2%	3.3%	31.7%	17.3%	15.9%	2.5%	2.7%	22.4%	12.5%	2.3%	2.1%	9.8%
S7	1	7.8%	9.6%	5.0%	1.0%	3.1%	9.4%	4.7%	3.5%	8.5%	5.1%	9.0%	4.7%	6.1%	6.4%
*p* < 0.001	2	12.7%	13.0%	12.6%	7.3%	3.1%	21.0%	9.0%	10.6%	17.6%	9.1%	15.7%	13.2%	16.3%	10.8%
V = 0.176	3	39.4%	40.2%	48.7%	48.0%	27.5%	31.8%	23.6%	44.4%	45.5%	45.1%	45.3%	41.1%	33.4%	63.2%
	4	26.7%	31.0%	28.8%	39.7%	40.1%	23.1%	33.3%	33.5%	22.8%	32.5%	24.4%	32.0%	35.0%	15.9%
	5	13.4%	6.1%	4.9%	4.0%	26.3%	14.7%	29.4%	8.1%	5.5%	8.1%	5.5%	9.1%	9.2%	3.7%
S8	1	7.5%	11.1%	5.8%	3.0%	7.3%	13.9%	5.5%	4.6%	13.9%	6.9%	10.2%	10.6%	14.3%	8.1%
*p* < 0.001	2	18.3%	11.5%	17.8%	19.0%	26.1%	23.5%	11.2%	19.2%	15.6%	8.5%	17.7%	14.5%	18.3%	13.5%
V = 0.191	3	49.1%	50.7%	57.5%	52.7%	48.2%	30.0%	29.2%	50.0%	52.4%	52.0%	52.6%	53.0%	47.8%	60.8%
	4	18.6%	21.6%	16.5%	25.0%	16.2%	23.1%	29.7%	23.7%	16.1%	27.8%	18.3%	17.2%	15.8%	13.5%
	5	6.5%	5.1%	2.4%	0.3%	2.2%	9.4%	24.3%	2.5%	2.1%	4.7%	1.2%	4.6%	3.8%	4.1%
S9	1	5.6%	5.6%	4.6%	2.7%	8.7%	16.5%	3.7%	3.3%	5.9%	5.5%	10.5%	3.1%	5.4%	6.4%
*p* < 0.001	2	5.6%	7.4%	7.7%	8.3%	14.0%	23.7%	7.2%	7.9%	8.0%	6.9%	10.2%	9.3%	6.8%	11.8%
V = 0.180	3	17.1%	36.2%	24.5%	36.0%	21.8%	31.2%	17.5%	25.4%	24.7%	29.3%	39.8%	30.9%	17.7%	56.8%
	4	33.2%	31.1%	36.2%	40.3%	32.8%	18.2%	31.0%	40.2%	35.9%	38.6%	29.7%	29.8%	30.8%	20.6%
	5	38.5%	19.7%	27.0%	12.7%	22.7%	10.4%	40.6%	23.3%	25.6%	19.7%	9.9%	26.9%	39.3%	4.4%
S10	1	8.1%	20.3%	8.0%	18.7%	3.6%	19.4%	4.0%	6.7%	12.5%	8.1%	11.3%	14.3%	19.5%	9.5%
*p* < 0.001	2	13.0%	19.0%	13.4%	31.0%	6.2%	26.3%	10.2%	14.8%	15.7%	11.0%	14.0%	19.1%	19.0%	14.5%
V = 0.197	3	45.0%	35.3%	48.1%	36.0%	35.3%	31.8%	27.5%	41.2%	52.8%	39.6%	43.9%	45.3%	42.4%	44.3%
	4	22.0%	19.7%	25.8%	13.3%	39.2%	16.1%	32.8%	31.3%	15.4%	31.5%	26.2%	15.5%	14.6%	29.1%
	5	11.8%	5.8%	4.7%	1.0%	15.7%	6.5%	25.5%	6.0%	3.6%	9.8%	4.7%	5.8%	4.5%	2.7%

^(1)^ S1. Entomophagy is a dietary practice that consists of the consumption of insects by humans; S2. Insects are considered a traditional food in my country; S3. There are thousands of species of insects that are consumed by humans in the world; S4. Consuming insects is characteristic of developing countries; S5. Insects are present in events related to religious rituals; S6. Insects are part of the gastronomic culture of most countries in the world; S7. In some countries, the tradition of eating insects is decreasing because of the “Westernization” of diets; S8. Insect consumption is seasonal, so it varies according to the time of the year; S9. There are obstacles to consumers’ acceptance of edible insects in Western countries; S10. Insects can be associated with traditional festivities and celebrations. ^(2)^ Statistics of Chi-square test: *p* = significance (considering a level of 5%), V = Cramer’s V coefficient. ^(3)^ Scale: 1 = Strongly disagree; 2 = Disagree; 3 = No opinion; 4 = Agree; and 5 = Strongly agree. ^(4)^ BR = Brazil, HR = Croatia, GR = Greece, LV = Latvia, LB = Lebanon, LT = Lithuania, MX = Mexico, PL = Poland, PT = Portugal, RO = Romania, RS = Serbia, SI = Slovenia, ES = Spain, and TR = Turkey.

**Table 6:** The solution obtained through factor analysis.

Factor	%VE ^(1)^	Item	Loading	Factor Name	Cronbach’s Alpha ^(2)^
F1	23.9%	S2. Insects are considered a traditional food in my country	0.453	CTEI (Culture and Tradition of EIs)	0.675
		S4. Consuming insects is characteristic of developing countries	0.557		
		S5. Insects are present in events related with religious rituals	0.684		
		S6. Insects are part of the gastronomic culture of most countries in the world	0.718		
		S8. Insect consumption is seasonal, so it varies according to the time of the year	0.463		
		S10. Insects can be associated with traditional festivities and celebrations	0.603		
F2	19.1%	S1. Entomophagy is a dietary practice that consists of the consumption of insects by humans	0.710	AEI (Acceptance of EIs)	0.614
		S3. There are thousands of species of insects that are consumed by humans in the world	0.461		
		S7. In some countries the tradition of eating insects is decreasing because of the “Westernization” of diets	0.573		
		S9. There are obstacles to consumers’ acceptance of edible insects in Western countries	0.755		

^(1)^ VE = Variance explained. ^(2)^ Cronbach’s alpha.

## Data Availability

The original contributions presented in this study are included in the article/Supplementary Materials. Further inquiries can be directed to the corresponding author.

## Supplementary Materials

The following supporting information can be downloaded at https://www.mdpi.com/article/10.3390/foods13213408/s1, Supplementary File: A full version of the questionnaire used.
