# Supplementary material for: Edible Insects: Consumption, Perceptions, Culture and Tradition Among Adult Citizens from 14 Countries
Source: Foods. 2024 Oct 25;13(21):3408. doi: 10.3390/foods13213408 (PMC11545776; doi:10.3390/foods13213408)
Supplement: Supplementary file 1 [file foods-13-03408-s001.zip › foods-3235700-supplementary.pdf]

EDIBLE INSECTS SURVEY

This presents the full version of the questions, as they were presented to the participants, after the introduction to the research to contextualize the work and after informed consent statement….

1. Demographic Data

1. **Age:** _____ years

2. **Sex:**

Female ☐ 1 Male ☐ 2 Don't want to answer ☐ 3

3. **Education level:**

Post-graduate education (master or doctorate) ☐ 1

Completed a university degree ☐ 2

No university degree ☐ 3

If you do not have a university degree, how many school years you have:____________ 3.a

4. **Living environment:**

Rural ☐ 1 Urban ☐ 2 Suburban ☐ 3

5. **Household income in relation to the average in your country:**

Much lower ☐ 1 Lower ☐ 2 Equal to the average ☐ 3 Higher ☐ 4 Much higher ☐ 5

6. **Professional activity / studies related to any of the following areas:**

	Yes	No
1. Food/Nutrition		
2. Agriculture		
3. Environment		
4. Biology		
5. Health-related activities		
6. Tourism		
7. Others		

2. Characterization of participants' habits

7. **How often do you eat in restaurants (please answer considering the pre-covid situation)?**

Never ☐ 1 Rarely (less than once per month) ☐ 2

Sporadically (between once per week and once per month) ☐ 3

Occasionally (about once per week) ☐ 4 Moderately (2 to 3 times per week) ☐ 5

Often (4 or more times per week) ☐ 6

8. **When you go to restaurants, what type of food you prefer (pre-covid)?**
(please choose 1 or 2 or a maximum of 3 options)

	My top choices
1. Traditional food from my country	
2. Ethnic food (typical from foreign countries)	
3. Regional specialities	
4. Gourmet food	
5. Fast-food	
6. Suggestion of the day/Done dish	
7. Healthy food	
8. Vegan/Vegetarian	
9. Grilled food/Barbecue	

9. **How often do you travel abroad (pre-covid)?**

Never ☐ 1 Rarely (about once per year) ☐ 2

Occasionally (about 2 times per year) ☐ 3 Often (3 or more times per year) ☐ 4

10. **When traveling abroad do you have a preference for the type of food you consume?**

Typical food of the country I am visiting ☐ 1

Food as similar as possible to my own country ☐ 2

International food (types of food commonly spread around the world) ☐ 3

No preference ☐ 4

11. **Do you consider Covi-19 changed your eating patterns?**

	Not changed1	Little changed2	Much changed3
1. Frequency of eating out			
2. Frequency of travelling abroad			
3. Frequency of ordering prepared food in the catering sector			
4. Type of food consumed			
5. Food shopping practices			
6. Food safety concerns			

12. **Have you ever eaten insects as culinary preparations, as snacks or other derived products?**

Yes ☐ 1 No ☐ 2 Don´t know/Don´t remember ☐ 3

13. **If you have never eaten insects, would you consider eating them?**

Definitely not ☐ 1 Maybe ☐ 2

Yes, but only derived foods that include insects (for example hamburger or biscuits) ☐ 3

Yes, whole insects and derived foods ☐ 4

13.a. In which circumstances you would consume? (you may choose more than one option)

Out of curiosity ☐ 1

If there is scarcity of food ☐ 2

To help preserve the planet ☐ 3

Because of the gastronomic characteristics ☐ 4

Because of the nutritional properties ☐ 5

14. **If you consume EI, how often do you eat insects as culinary preparations, as snacks or other derived products?**

About one time per year ☐ 1 About 2 to 3 times per year ☐ 2

About one time per month ☐ 3 About one time per week ☐ 4 Two or more time per week ☐ 5

15. **What comes to your mind when you hear about edible insects? Please use up to 5 words or small expressions that you associate with edible insects.**

1)____

2)____

3)____

4)____

5)____

D1. Culture & Tradition

16. Please provide your opinion on the following information (Scale: 1 = Strongly disagree, 2 = Disagree, 3 = Indifferent, 4 = Agree, 5 = Strongly agree).

	Strongly Disagree	Disagree	No opinion	Agree	Strongly Agree
1. Entomophagy is a dietary practice that consists in the consumption of insects by humans	☐ 1	☐ 2	☐ 3	☐ 4	☐ 5
2. Insects are considered a traditional food in my country	☐ 1	☐ 2	☐ 3	☐ 4	☐ 5
3. There are thousands of species of insects that are consumed by humans in the world	☐ 1	☐ 2	☐ 3	☐ 4	☐ 5
4. Consuming insects is characteristic of developing countries	☐ 1	☐ 2	☐ 3	☐ 4	☐ 5
5. Insects are present in events related with religious rituals	☐ 1	☐ 2	☐ 3	☐ 4	☐ 5
6. Insects are part of the gastronomic culture of most countries in the world	☐ 1	☐ 2	☐ 3	☐ 4	☐ 5
7. In some countries the tradition of eating insects is decreasing because of the "Westernization" of diets	☐ 1	☐ 2	☐ 3	☐ 4	☐ 5
8. Insect consumption is seasonal, so it varies according to the time of the year	☐ 1	☐ 2	☐ 3	☐ 4	☐ 5
9. There are obstacles to consumers' acceptance of edible insects in Western countries	☐ 1	☐ 2	☐ 3	☐ 4	☐ 5
10. Insects can be associated with traditional festivities and celebrations	☐ 1	☐ 2	☐ 3	☐ 4	☐ 5

D2. Gastronomic innovation & Gourmet kitchen

17. **Please provide your opinion on the following information (Scale: 1 = Strongly disagree, 2 = Disagree, 3 = Indifferent, 4 = Agree, 5 = Strongly agree).**

	Strongly Disagree	Disagree	No opinion	Agree	Strongly Agree
1. Insects are considered as exotic foods	☐ 1	☐ 2	☐ 3	☐ 4	☐ 5
2. Insects are traded as treats/delicacies	☐ 1	☐ 2	☐ 3	☐ 4	☐ 5
3. Insects are not suitable for human consumption	☐ 1	☐ 2	☐ 3	☐ 4	☐ 5
4. Insects are associated with taboos and food neophobia (not wanting to eat unfamiliar foods)	☐ 1	☐ 2	☐ 3	☐ 4	☐ 5
5. Some gourmet restaurants use edible insects in their culinary preparations	☐ 1	☐ 2	☐ 3	☐ 4	☐ 5
6. Insects are present in culinary events and gastronomic shows	☐ 1	☐ 2	☐ 3	☐ 4	☐ 5
7. Insects are recommended by some recognized chefs	☐ 1	☐ 2	☐ 3	☐ 4	☐ 5
8. Chefs contribute to the popularization of insects into gastronomy in Western countries	☐ 1	☐ 2	☐ 3	☐ 4	☐ 5
9. Culinary education favours overall liking for innovative insect based products	☐ 1	☐ 2	☐ 3	☐ 4	☐ 5

D3. Environment & Sustainability

18. Please provide your opinion on the following information (Scale: 1 = Strongly disagree, 2 = Disagree, 3 = Indifferent, 4 = Agree, 5 = Strongly agree).

	Strongly Disagree	Disagree	No opinion	Agree	Strongly Agree
1. Insects are a more sustainable alternative when compared to other sources of animal protein	☐ 1	☐ 2	☐ 3	☐ 4	☐ 5
2. Insect production for human consumption emits much less greenhouse gases than beef production	☐ 1	☐ 2	☐ 3	☐ 4	☐ 5
3. Insects efficiently convert organic matter into protein	☐ 1	☐ 2	☐ 3	☐ 4	☐ 5
4. The production of insect protein uses considerably less feed than cow protein	☐ 1	☐ 2	☐ 3	☐ 4	☐ 5
5. Insects are a possibility to respond to the growing world demand for protein	☐ 1	☐ 2	☐ 3	☐ 4	☐ 5
6. The production of chicken protein requires much less water than insect protein	☐ 1	☐ 2	☐ 3	☐ 4	☐ 5
7. The ecological footprint (impact) of insects is smaller when compared to other animal proteins	☐ 1	☐ 2	☐ 3	☐ 4	☐ 5
8. The production of insect protein requires much more area than pig protein	☐ 1	☐ 2	☐ 3	☐ 4	☐ 5
9. Insects are collected as a means of pest control for some cultivated crops	☐ 1	☐ 2	☐ 3	☐ 4	☐ 5
10. Loss of biodiversity is lower with insect production compared to other animal food production	☐ 1	☐ 2	☐ 3	☐ 4	☐ 5
11. Energy input needed for production of insect protein is lower than for the production of other proteins from animal origin	☐ 1	☐ 2	☐ 3	☐ 4	☐ 5

D4. Economic & Social aspects

19. Please provide your opinion on the following information (Scale: 1 = Strongly disagree, 2 = Disagree, 3 = Indifferent, 4 = Agree, 5 = Strongly agree).

	Strongly Disagree	Disagree	No opinion	Agree	Strongly Agree
1. Insect production can contribute to increase the income of families in low income areas	☐ 1	☐ 2	☐ 3	☐ 4	☐ 5
2. Insects provide protein foods at cheap prices	☐ 1	☐ 2	☐ 3	☐ 4	☐ 5
3. The market of edible insects is expected to decline in the future	☐ 1	☐ 2	☐ 3	☐ 4	☐ 5
4. Presently, the Asia-Pacific and Latin America areas account for more than half of the edible insects market	☐ 1	☐ 2	☐ 3	☐ 4	☐ 5
5. In some countries insect farming is becoming a key factor to fight against rural poverty	☐ 1	☐ 2	☐ 3	☐ 4	☐ 5
6. The income generated from insects can be affected by market fluctuations in price derived from availability	☐ 1	☐ 2	☐ 3	☐ 4	☐ 5

D5. Commercialization & Marketing

20. Please provide your opinion on the following information (Scale: 1 = Strongly disagree, 2 = Disagree, 3 = Indifferent, 4 = Agree, 5 = Strongly agree).

	Strongly Disagree	Disagree	No opinion	Agree	Strongly Agree
1. Edible insects are difficult to find on sale on street markets	☐ 1	☐ 2	☐ 3	☐ 4	☐ 5
2. Edible insects are easy to find on sale in supermarkets	☐ 1	☐ 2	☐ 3	☐ 4	☐ 5
3. Edible insects are on sale only on specialized shops	☐ 1	☐ 2	☐ 3	☐ 4	☐ 5
4. The level of knowledge influences the willingness to purchase insect food	☐ 1	☐ 2	☐ 3	☐ 4	☐ 5
5. Price is among the motivations to consume insect foods	☐ 1	☐ 2	☐ 3	☐ 4	☐ 5
6. The consumption of insects and derived foods depends on availability	☐ 1	☐ 2	☐ 3	☐ 4	☐ 5
7. Personalities/influencers can lead people to consume insects	☐ 1	☐ 2	☐ 3	☐ 4	☐ 5
8. Insect consumption is independent of marketing campaigns	☐ 1	☐ 2	☐ 3	☐ 4	☐ 5

D6. Nutritional aspects

21. Please provide your opinion on the following information (Scale: 1 = Strongly disagree, 2 = Disagree, 3 = Indifferent, 4 = Agree, 5 = Strongly agree).

	Strongly Disagree	Disagree	No opinion	Agree	Strongly Agree
1. Insects have poor nutritional value	☐ 1	☐ 2	☐ 3	☐ 4	☐ 5
2. Insects are a good source of energy	☐ 1	☐ 2	☐ 3	☐ 4	☐ 5
3. Insects have high protein content	☐ 1	☐ 2	☐ 3	☐ 4	☐ 5
4. Insect proteins are of poorer quality compared to other animal species	☐ 1	☐ 2	☐ 3	☐ 4	☐ 5
5. Insects provide essential amino acids necessary for humans	☐ 1	☐ 2	☐ 3	☐ 4	☐ 5
6. Insects contain group B vitamins	☐ 1	☐ 2	☐ 3	☐ 4	☐ 5
7. Insects contain dietary fibre	☐ 1	☐ 2	☐ 3	☐ 4	☐ 5
8. Insects contain minerals of nutritional interest, such as calcium, iron, and magnesium	☐ 1	☐ 2	☐ 3	☐ 4	☐ 5
9. Insects contain fat, including unsaturated fatty acids	☐ 1	☐ 2	☐ 3	☐ 4	☐ 5
10. Insects contain anti-nutrients, such as oxalates and phytic acid	☐ 1	☐ 2	☐ 3	☐ 4	☐ 5

D7. Health effects

22. Please provide your opinion on the following information (Scale: 1 = Strongly disagree, 2 = Disagree, 3 = Indifferent, 4 = Agree, 5 = Strongly agree).

	Strongly Disagree	Disagree	No opinion	Agree	Strongly Agree
1. There are appropriate regulations to guarantee food safety of edible insects	☐ 1	☐ 2	☐ 3	☐ 4	☐ 5
2. Insects are used by some people in traditional medicine	☐ 1	☐ 2	☐ 3	☐ 4	☐ 5
3. Eating insects poses a substantial risk to human health	☐ 1	☐ 2	☐ 3	☐ 4	☐ 5
4. Industrial processed insect products are hygienic and safe	☐ 1	☐ 2	☐ 3	☐ 4	☐ 5
5. Insects and insect based foods are often infected by pathogens and parasites	☐ 1	☐ 2	☐ 3	☐ 4	☐ 5
6. Insects collected from the wild may be contaminated with pesticide residues	☐ 1	☐ 2	☐ 3	☐ 4	☐ 5
7. In certain countries insects are approved officially for therapeutic treatment	☐ 1	☐ 2	☐ 3	☐ 4	☐ 5
8. Insects contain bioactive compounds beneficial to human health	☐ 1	☐ 2	☐ 3	☐ 4	☐ 5
9. Insects are potential sources of allergens	☐ 1	☐ 2	☐ 3	☐ 4	☐ 5
10. Aflatoxins, which are carcinogens, can be present in insects	☐ 1	☐ 2	☐ 3	☐ 4	☐ 5

Thank you for your collaboration.
